# Application of pattern recognition tools for classifying acute coronary syndrome: an integrated medical modeling

**DOI:** 10.1186/1742-4682-10-57

**Published:** 2013-09-18

**Authors:** Nader Salari, Shamarina Shohaimi, Farid Najafi, Meenakshii Nallappan, Isthrinayagy Karishnarajah

**Affiliations:** 1 Department of Biology, Faculty of Science, University Putra Malaysia, Serdang, Selangor, Malaysia; 2 Department of Biostatistics and Epidemiology, School of Public Health, Kermanshah University of Medical Sciences, Kermanshah, Iran; 3 Department of Mathematics, Faculty of Science, University Putra Malaysia, Serdang, , Selangor, Malaysia

**Keywords:** Acute coronary syndrome, Artificial intelligence, Clinical decision support systems, Classification, Diagnosis

## Abstract

**Objective:**

The classification of Acute Coronary Syndrome (ACS), using artificial intelligence (AI), has recently drawn the attention of the medical researchers. Using this approach, patients with myocardial infarction can be differentiated from those with unstable angina. The present study aims to develop an integrated model, based on the feature selection and classification, for the automatic classification of ACS.

**Methods:**

A dataset containing medical records of 809 patients suspected to suffer from ACS was used. For each subject, 266 clinical factors were collected. At first, a feature selection was performed based on interviews with 20 cardiologists; thereby 40 seminal features for classifying ACS were selected. Next, a feature selection algorithm was also applied to detect a subset of the features with the best classification accuracy. As a result, the feature numbers considerably reduced to only seven. Lastly, based on the seven selected features, eight various common pattern recognition tools for classification of ACS were used.

**Results:**

The performance of the aforementioned classifiers was compared based on their accuracy computed from their confusion matrices. Among these methods, the multi-layer perceptron showed the best performance with the 83.2% accuracy.

**Conclusion:**

The results reveal that an integrated AI-based feature selection and classification approach is an effective method for the early and accurate classification of ACS and ultimately a timely diagnosis and treatment of this disease.

## Background

Acute coronary syndrome (ACS) is caused by insufficient blood supply to the heart muscle which itself is mostly caused by the rupture of an atherosclerotic plaque resulting in a partial or complete blockage of coronary arteries [1]. ACS is generally classified into three coronary arteries-related conditions: ST elevation myocardial infarction (STEMI), non ST elevation myocardial infarction (NSTEMI), and unstable angina (UA) [2].

ACS is one of the most common problems among patient admitted to the emergency departments. According to a conservative estimate, at least 6 million patients present to emergency departments with suspected ACS each year in the United States. In spite of the high frequency of this presentation, accurate diagnosis of ACS remains still challenging and requires a novel approach [3]. It is estimated that there is a 2–5% chance of misdiagnosis among patients with suspected ACS, which is potentially life-threatening [4]. Therefore, developing an automatic diagnostic system, based on the available clinical data, can be an effective solution for reducing this risk.

Past literature has attempted to identify automatic predictions that classify the three types of ACS using pattern recognition or machine learning approaches [4]. These methods have been developed on the basis of major clinical features of the ACS such as: ECG and Troponin level. Due to the overwhelming number of available features for each patient such as: age, weight, ECG, blood pressure, and medical history, it is quite challenging to select a subset of features that reliably contribute the most to the classification of ACS subtypes.

**Figure 1:**
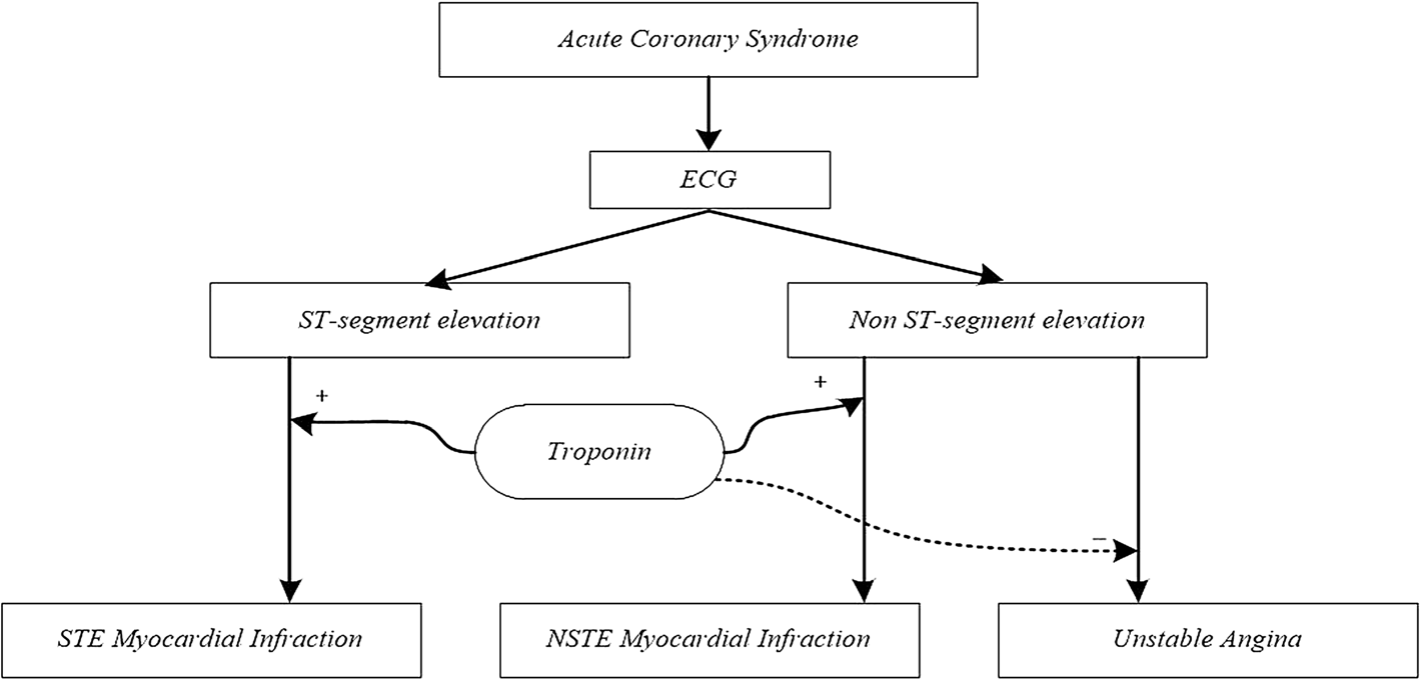
A diagnostic algorithm of classification of ACS based on ECG changes and Troponin level.

Pattern recognition algorithms have been widely used to classify UA from MI [5]. While such studies have used various features, they have not classified ACS based on both the ECG findings and Troponin level. On the other hand, according to the World Health Organization, the diagnosis criteria of MI are the combinations of at least two of these three major factors: (1) typical clinical manifestations of infarction (i.e.; chest pain), (2) change in marker’s pattern and (3) a typical ECG pattern involving the ST-segment changes on ECG [6]. In the current study, both the ECG findings and Troponin level have been used for ACS classification. Figure 1 demonstrates the algorithm for diagnosis of patients suspected to have ACS.

Artificial neural networks (ANNs) are powerful and effective tools for the classification and prediction of diseases. These methods are capable of constructing a nonlinear mapping between the input and output. Several studies have used ANNs for classification of ACS data. Harrison et al. [4] used Multilayer Perceptron (MLP), which is a common type of ANNs, for differentiating UA from MI by selecting 13 out of 40 features. They have achieved a good predictive performance using ECG findings while excluding Troponin level.

Similar to Harrison et al.’s study, Forberg et al. [7] considered only the ECG information for classifying ACS patients. In their study, the performance of ANNs and logistic regression were compared to the physicians’ decisions. The results showed a relatively higher efficacy of logistic regression as compared to the ANN. Moreover, Colak et al. [8] showed a good efficacy of eight learning algorithms for ANNs in detecting ACS based on clinical data. This being said, one of the main limitations of employing ANNs for classification of ACS is the lack of explanations of the findings. This issue was addressed by comparing artificial datasets with real clinically recorded ACS data [7-9].

Other artificial intelligence expert systems have also been used in the detection and classification of heart disease. For instance, Adeli et al. [10] proposed a fuzzy expert system for classifying patients into five different groups: healthy, typical angina, atypical angina, non-angina, and asymptomatic. This system also uses clinical data such as ECG and blood indices for clinical decision making. The results obtained this way were comparable to the diagnosis of clinicians. Overall, it seems that all of the current ACS classification methods have been designed to discriminate MI from UA patients.

The present study aims to improve and extend the classification approach to discriminate among all three types of ACS: UA, NSTEMI, and STEMI. The classification methods that are introduced in this study were selected from eight well-known pattern recognition algorithms such as the Generalized Linear Models (GLMs), Adaptive Network Fuzzy Interface System (ANFIS), radial basis functions (RBF), k-nearest neighbor (k-NN), MLP, Naive Bayes, iterative dichotomiser-3 (ID3), and Baggin-ID3. Moreover, a feature selection algorithm based on the k-NN classifier was used to remove the redundant features of the dataset thereby increasing the efficacy of the proposed classification approach.

## Methods

### Dataset technical information

For patients admitted with a tentative diagnosis of ACS to Imam Ali Hospital (i.e. the main center for cardiovascular care in Kermanshah, Iran) was completed the Euro Heart Survey on ACS. This questionnaire was designed by the European Society of Cardiology and has shown reliability and consistency: it was first conducted in 25 countries (in 2000–2001) and again in 32 European countries [11]. All patients admitted with a tentative diagnosis of ACS to Imam Ali hospital in during 2010–2011 were included. According to the standard protocol of European ACS registry, all patients with unstable angina as well as those suspected of acute myocardial infarction were differentiated using elevating the cardiac markers: troponin, CK, and CK-MB and more than one of the suggestive characteristics such as (i) symptoms of myocardial ischemia, (ii) the development of new Q waves, and (iii) ST-T abnormalities suggestive for ischemic origin [12]. A total number of 809 patients were enrolled in this study. They were divided into four different groups based on the ACS including: STEMI, NSTEMI, UA, and other. Similar to with previous studies, follow up data were collected within a year for every patients. The forms were completed by the attending physician. A data collection officer reviewed and checked each form for the probable missing data.

For each subject, 266 clinical factors were collected consisting of both numeric and nominal features. Based on interviews with cardiologists as well as the references in the literature, 40 seminal attributes for classifying ACS were selected. These factors along with the values and data types are shown in Table 1.

**Table 1:** Detailed description of recorded clinical features of our ACS data

**Label**	**Feature name**	**Value**	**Scale**
1	Sex	Male=1, Female=2	Nominal
2	Age	[-1,1]	Ratio
3	Living place (rural or urban)	Urban=1, Rural=2	Nominal
4	Body Mass Index	[-1,1]	Ratio
5	History of prior myocardial infarction	Absence=1, Presence=2	Nominal
6	History of prior angina pectoris	Absence=1, Presence=2	Nominal
7	History of congestive heart failure	Absence=1, Presence=2	Nominal
8	History of stroke	Absence=1, Presence=2	Nominal
9	History of chronic renal failure	Absence=1, Presence=2	Nominal
10	History of chronic lung disease	Absence=1, Presence=2	Nominal
11	Prioritize PCI	Absence=1, Presence=2	Nominal
12	Prior CABG	Absence=1, Presence=2	Nominal
13	Smoking status	Never=1, Former=2, Current=3	Ordinal
14	Diabetes mellitus	Non-diabetic=1, Newly diagnosed =2, Diabetic (dietary control) =3, Diabetic (oral medication) =4, Diabetic (oral MEDs + insulin) =5, Diabetic (insulin) =6	Ordinal
15	History of hypertension	Absence=1, Presence=2	Nominal
16	History of hypercholesterolemia	Absence=1, Presence=2	Nominal
17	Family history of CAD	Absence=1, Presence=2	Nominal
18	Chronic Home MEDs: Aspirin	Absence=1, Presence=2	Nominal
19	Chronic Home MEDs: Other antiplatelet	None=1, Other antiplatelet agent=2, Clopidogrel=3 , Ticlopidine=4	Ordinal
20	Chronic Home MEDs: Anticoagulants	Absence=1, Presence=2	Nominal
21	Chronic Home MEDs: Beta-blockers	Absence=1, Presence=2	Nominal
22	Chronic Home MEDs: ACE inhibitors	Absence=1, Presence=2	Nominal
23	Chronic Home MEDs: Angiotensin II RB	Absence=1, Presence=2	Nominal
24	Chronic Home MEDs: Statins	Absence=1, Presence=2	Nominal
25	Chronic Home MEDs: Non-statin lipid low. Agents	None= 1 , Other non-statin=2, Fibrates=3, Ezitimibe=4	Ordinal
26	Chronic Home MEDs: Calcium channel blockers	Absence=1, Presence=2	Nominal
27	Chronic Home MEDs: Calcium channel blockers	Absence=1, Presence=2	Nominal
28	Predominantly presenting symptom	Asymptomatic=1, Fatigue=2, Chest pain=3, Dyspnoea=4, Other symptoms=5, Syncope=6, Cardiac arrest-Aborted sudden death= 7	Ordinal
29	Heart rate	[-1,1]	Ratio
30	Systolic blood pressure	[-1,1]	Ratio
31	Troponin I elevated	Absence=-1, Presence=1	Nominal
32	CKMB mass elevated	Absence=-1, Presence=1	Nominal
33	Total Cholesterol value	[-1,1]	Ratio
34	Serum creatinine value	[-1,1]	Ratio
35	Glucose value	[-1,1]	Ratio
36	Hemoglobin value	[-1,1]	Ratio
37	Killip class	Class I=1, Class II=2, Class III=3, Class IV= 4	Ordinal
38	ECG rhythm	Sinus rhythm=1, Atrial fibrillation=2, Pacemaker=3, Other=4	Ordinal
39	ECG QRS annotation	Normal=1, RBBB=2, LBBB=3, Other=4	Ordinal
40	ECG STT changes	Normal=1, Other=2, ST depression=3, ST elevation=4	Ordinal

In the current study, we utilized both numerical and categorical variables. The numerical variables were re-scaled to [-1, 1], by min-max normalization technique. The re-scaling was carried out in order to deal with the inconsistencies between different features. This transformation technique has two important advantages. The main advantage of min-max normalization lies in its ability to rescale the values so that they fall within a predetermined range. In addition, it reserves the relationships between the initial data [13].

### Pattern recognition methods

Different classification methods for modeling ACS data were applied to achieve different classifiers for classifying of new subjects. The classifying performance of these classifiers was compared with respect to their performance in classification prediction. These classification methods, described in the following section, were selected from different tools including GLMs, ANFIS, RBF, k-NN, MLP, Naive Bayes, ID3, and Bgging-ID3.

### Generalized linear models

GLMs are powerful methods in applied statistical, which generalizes the ordinary linear models [14]. In this approach, the output variable y is modeled by linear combination of input variables *x*_*i*_ (features):

(1) $$y=\sum_{i}b_{i}.x_{i}$$

Assuming a probability function for the variables, the statistical mean of the output may have a certain link function as shown in Table 2[14]. Finally, using the generalized least square method, the unknown parameters of the model are estimated.

**Table 2:** Different probability distribution and their corresponding link function used in GLMs

**Distribution**	**Link function**
**Normal**	*μ* = *Xb*
**Inverse Gaussian**	*μ*^*-2*^ = *Xb*
**Poisson**	Log (*μ*) = *Xb*
**Gamma**	*μ*^*-1*^ = *Xb*

### k-nearest neighbor

k-NN is known as a very simple and popular classification algorithm. k-NN classifier, for each new sample, finds the k neighbors nearest to the new sample from the training data. Euclidean distance or correlation measure is usually used to find these neighbors. The new sample is then assigned to the class which has the most abundance in the neighboring samples [15].

### Multilayer perceptron

MLPs are the most common structures of the ANNs, which can be used for both regression and classification problems. MLP is known as a feed forward neural network trained by Back Propagation algorithm with one or more layers between input and output layer. Feed forward means that the data flows in one direction from the input to the output layer. In addition, back-propagation refers to the method for computing the gradient of the error function with respect to the weights for a feed-forward network. MLP consists of neurons which are connected to each other with some weights. Each neuron sums its inputs from the neurons of the previous layer and passes the sum through a sigmoidal or S-shaped activation function [16]. It has been shown that an MLP with one hidden layer can produce enough complexity to map any input and output data [17].

### Radial basis functions

RBF networks can be interpreted as feed-forward networks consisting of an input layer, a hidden layer and an output layer [17]. In hidden layer each neuron consists of an activation function which is a radial basis kernel function (typically a Gaussian function). The output of the radial basis activation function is inversely proportional to the distance between its input and the center of the neuron. Although the structure of RBF networks resembles that of MLPs, their input–output mappings and training algorithms are basically different. RBFs are typically trained using a hybrid algorithm in two steps [18]. In the first step, the hidden layer is trained (i.e. determining the radial basis centers and the spreads) by an unsupervised learning method. In the next step, the output layer is trained (i.e. Predicting the target outputs) by a supervised learning method.

### Adaptive network fuzzy interface system

ANFIS is an integrated neural-fuzzy network based on neural network and fuzzy rules [19]. The structure of this network is similar to an MLP; however, its neurons have different functions. Indeed, it is a special case of an adaptive network.

In adaptive networks there are two types of neurons (nodes): (i) fixed nodes which perform simple addition and multiplication, and (ii) adaptive neurons which have adaptive parameters and need to be estimated based on the input and the output data. In effect, this approach is generally a regression method which is used as a classifier in the classification problem. Thus, a tremendous performance from this classifier should not be expected.

### Naive Bayes

Applying Naive Bayes classifier, each new sample is assigned to the most probable class based on the Bayes decision making. The probability functions of the classes are empirically estimated from the training data. In spite of the low computational complexity, this method has a relatively high performance [20].

### Iterative dichotomiser-3

Decision trees are powerful and effective approaches to create a classification model. This method is a flow-chart-like tree structure, where a tree is constructed by the “if-then” rules (i.e. A logical sequence of questions) extracted from the training data [9,21]. A new case can be classified by starting at the root of the tree and moving through it until a leaf is encountered. Decision trees have become one of the most widely applied methods among numerous classification approaches, because these are white box models with easy-to-interpret results. In addition, its construction does not need any domain knowledge or parameter setting and thus is appropriate for exploratory knowledge discovery [22,23]. ID3 is one of the major algorithms of decision tree which was used in this study.

### Bagging-ID3

Bagging (Bootstrap aggregating) is a popular approach proposed by Bremen [24,25] which is considered as an ensemble meta-algorithm to build classification models. This resampling-based technique can be incorporated into various classification algorithms or regression methods. This incorporation resulted in reducing the variance associated with the prediction models, and thereby improves the predictive performance of these models. Bagging consists of creating numerous bootstrap replicates of the learning set by drawing “B” simple random samples with replacement (bootstrap samples) from the learning set and using these as new learning sets. Then, the considered prediction model is applied to each “B” bootstrap sample (i.e. new learning sets). To construct the final model, the results (i.e. the “B” built models) subsequently are combined into an ensemble by averaging for regression and simple voting for classification [26-28].

In fact, the true strength of bagging approach is for unstable models, such as decision trees and neural networks. Unstable models are sensitive to small alterations in the dataset. Hence, training the same model on two slightly different training sets might result in substantially different models (i.e. The models with different parameters similar overall accuracies) [25,29]. Thus, bagging can be a good solution to overcome this problem. In order to overcome unstably of the ID3, classifier bagging can be incorporated into the ID3 and emerge Bagging-ID3 classifier.

### Feature selection

Feature selection is one of the important steps in a classification problem. In reality, there are usually many redundant features which do not have any contributions in discriminating classes. Moreover, redundant features increase the complexity of the classification algorithm. Thus, they may have an effect on the performance of the model and may decrease its accuracy as well.

There are two main approaches for performing dimensionality reduction of high dimensional data [18]. The first approach is feature extraction, which focuses on transforming the existing features into a lower dimensional space. Most feature extraction methods have been based on two major linear techniques: principal components analysis and Fisher’s linear discriminant analysis [16,18]. Although they can considerably reduce the number of features, the resulting new features are still a function of the initial features. Thereby, it is usually impossible to find a physical interpretation of these new features. The second approach is feature selection, which is also called feature subset selection in the pattern recognition literature. The goal of the feature selection approach is to find an “optimal” subset of features that maximizes information content or predictive accuracy.

In classification problems, feature selection finds a subset of features which generates the best discrimination among classes. Some discrimination indexes can be used for this purpose [4]. Since these indexes are easy to calculate, the whole subset searching procedure can be performed quickly. However, these indexes are independent of the classification algorithm and thus the selected subset may not be the best choice for the classification task. In proposing model by Peng and Jinjin [30], a genetic algorithm-based strategy for feature selection in heart disease classification is used. In this approach, the optimal subset of features is found using GA.

We utilized a procedure for feature selection to yield a subset of features with the best classification accuracy. To this end, a k-NN classifier for the classification and the elimination algorithm for feature selection was employed:

0- Set k to 0

1. k ← k+1 and S ←{f_1_, f_40_}

2. For i = 1, l where l is the size of current selected subset, S, do the following steps

a. S_i_ = S-[31]

a. Perform the classification task with current Si and k and repeat it 100 times using different randomly selected training and test data

a. acc_i_ = average of all accuracy values from previous step

3. Select the best subset: S ←S_i_*, where i* = argmax acc_i_

4. Go back to 2 until l = 1

5. Go back to 1 until k = 13

6. Ending this algorithm is optimized for both the selected subset of features and the parameter k of k-NN classifier.

### Performance assessment

#### ***Model validation***

Model validation is one of the most important steps in the model building process [32]. Cross-validation is the most popular resampling-based model validation method [33,34]. The various types of cross-validation method include: data holdout, repeated random sub-sampling, k-fold, and leave-one-out [32,33]. In the current study, repeated random sub-sampling cross-validation method was adopted for the model validation. The dataset was split into two sets of training and test (i.e. two-way data splitting method). The training set was used to find the model’s parameters and the test set was used to evaluate the generalizability performance of the final model. The process of train–test was repeated 50 to 1000 times (i.e. adopted according to the used model) using randomly selected training and test sets. Finally, the estimate of the overall error rate was derived by averaging all the separate error rate estimates produced from different iterations.

Cross-validation method can help avoid two important issues in pattern recognition problems: (i) overfitting of the final model (i.e. the final model is unable to generalize unseen data) and (ii) the error rate estimate will be overly optimistic (i.e. lower than the true error rate) [31]. It should be noted that in order to select the model and estimate the error rate simultaneously, three-way data splits technique should be applied during the cross validation process [31,32]. In other words, the data should be divided into three disjoint sets namely training, validation, and test sets.

In this procedure, the training set was used for learning, i.e. to optimize the tuning parameters of the model (e.g. In MLP, in order to determine the optimal weights and the bias with the back-propagation rule). The validation set was used to optimize the regularization parameters of the model (e.g. In MLP, in order to determine the optimal number of hidden units and a stopping point of the algorithm). The test set was used only to estimate the error rate of the final model (fully-tuned model). After assessing the final model based on the test set, the model must not be further tuned. Table 3 presents the data splitting method and also the number of repetitions (based on model’s computation complexity) for each classifier method.

**Table 3:** The used data splitting methods and number of repetitions for each classifier method

**NO.**	**Classifier**	**Data splitting method**	**Number of repetitions**
1	ANFIS	Three-way	50
2	MLP	Three-way	100
3	RBF	Three-way	1000
4	Bagging ID3	Three-way	1000
5	ID3	Two-way	1000
6	GLM	Two-way	1000
7	k-NN	Two-way	1000
8	Naive Bayes	Two-way	1000

#### ***Model performance evaluation criteria***

There are a number of criteria used to quantify the performance of a model [32,35]. The performance of the final model can be evaluated by estimating the model accuracy rate. The evaluation operation is generally performed by comparing the predicted class labels with the actual class labels.

A matrix called Confusion Matrix (CM) is used to show the performance of a model for certain problems [32]. If we have C classes, the CM is C×C matrix whose elements *CM*_*ij*_ show the misclassified number of samples from class I into class j. Therefore, the rows and columns of this matrix show the actual and predicted class labels, respectively. In Table 4, part A shows a summing CM, the underlined number (i.e. corresponding to the predicted class of 4 and actual classes of 3) indicates that there are 11 samples from class 3 misclassified as class 4. Consequently, the smaller off-diagonal elements are the better performance of the classifier. When there are only two classes, other indexes such as sensitivity and specificity are usually used instead of CM.

**Table 4:** An example of CM, APM, and CPM

**Actual class**	**Predicted class**			
	**Class1**	**Class2**	**Class3**	**Class4**
**A: CM**				
**Class1**	1	0	1	11
**Class2**	0	49	1	5
**Class3**	0	6	12	11
**Class4**	3	0	7	95
**B: APM**				
**Class1**	0.08	0.00	0.08	0.85
**Class2**	0.00	0.89	0.02	0.09
**Class3**	0.00	0.21	0.41	0.38
**Class4**	0.02	0.00	0.07	0.91
**C: CPM**				
**Class1**	0.01	0.00	0.00	0.01
**Class2**	0.00	0.83	0.18	0.00
**Class3**	0.02	0.01	0.19	0.02
**Class4**	0.97	0.16	0.63	0.98

A common index for evaluating the performance of a classifier is accurate which is calculated from the CM as follows:

(2) $$\text{Acc}=\frac{\sum_{i}C.M_{\mathrm{ii}}}{\sum_{i}\sum_{j}C.M_{\mathrm{ij}}}$$

If the elements of this matrix are divided by the actual number of each class (i.e. which is equal to the sum of each row), each element (*i,j*) of the resulting matrix would show the prediction probability $p\;\left(c_{i}^{*}|c_{i}\right)$ This conditional probability indicates the probability that the classifier assigns a sample of class *C*_*i*_ to class $\;c_{j}^{*}$. Therefore, $p\;\left(c_{i}^{*}|c_{i}\right)$ is the accuracy of the classifier for class *C*_*i*_. This matrix is called accuracy probability matrix (APM). Another useful probability measure is $p\;\left(c_{i}|c_{j}^{*}\right)$ which indicates that the probability of a sample classified as $c_{j}^{*}$ actually belongs to *C*_*i*_. Similarly, $p\;\left(c_{i}|c_{j}^{*}\right)$ shows the classification correctness of the classification *C*_*i*_ called correctness probability matrix (CPM) whose elements can be calculated simply from APM by the following relation:

(3) $$p\;\left(c_{i}|c_{j}^{*}\right)=\frac{p\;\left(c_{j}^{*}|c_{i}\right).p\left(c_{i}\right)}{\sum_{k}p\;\left(c_{j}^{*}|c_{k}\right).p\left(c_{k}\right)}$$

where *p*(*c*_*i*_) is the prior probability of the class *C*_*i*_. The off-diagonal elements of these matrixes, CM, APM, or CPM, for the perfect ideal classifier are zero. The APM and CPM corresponded with CM is presented in Table 4, part B and C.

## Results and discussion

Table 5 shows the distribution of ACS subtypes (Classes’ names) in the ACS dataset.

**Table 5:** Class sample distribution in the ACS dataset

**Class label**	**Class name**	**Frequency**	**Percent**
1	STEMI	224	27.69
2	NSTEMI	128	15.82
3	UA	417	51.55
4	Other	40	4.94
	Total	809	100.0

**Figure 2:**
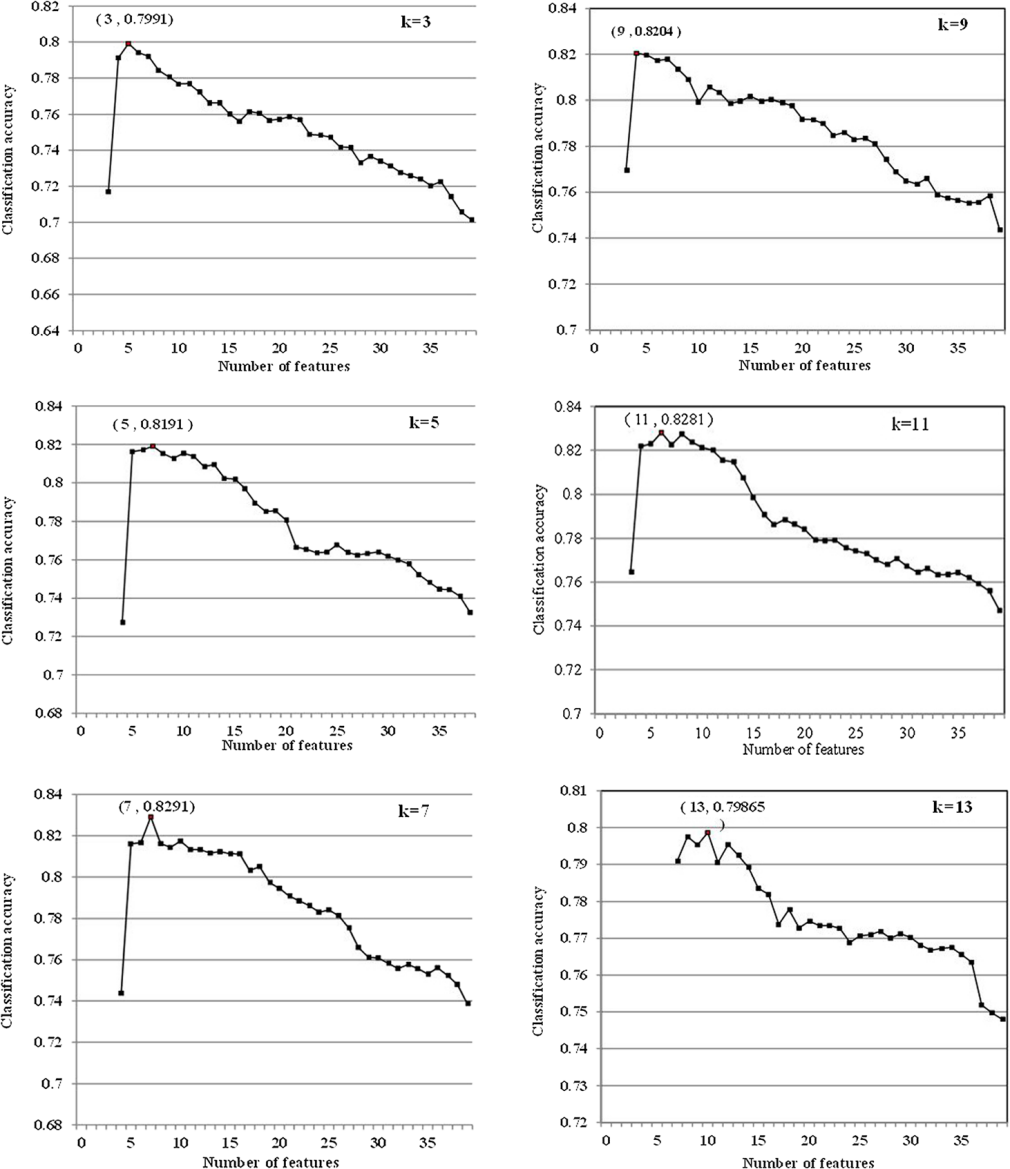
Classification accuracy plots versus the number of selected features in k-NN classifier for different odd values of k (k=3, 5, 7, 9, 11 and 13).

The feature selection algorithm was implemented for different odd values of k. The accuracy plots are shown in Figure 2 for the different odd values of k. As displayed in Table 6, k = 7 provided the best accuracy for the seven features. As it was expected, Troponin and ECG were presented with these selected features which can be a validation of feature selection algorithms [6]. It should be noted that, a limitation of the study (as in most medical studies) was the relatively limited sample size problem. Accordingly, in this study, all data were used in the feature selection process.

**Table 6:** Final selected features resulted from the feature selection algorithm

**Label**	**Variable name**
4	Body Mass Index
**10**	History of Chronic lung disease
**27**	Chronic Home MEDs: Calcium channel blockers
30	Systolic blood pressure
**31**	Troponin I elevated
36	Hemoglobin value
40	ECG STT changes

After obtaining the optimal features for the classification tasks, these features in all the classifiers were used and their performances were compared with APM and overall classification accuracy values. The resulted APMs from the GLM method with four different distributions are presented in Table 7. The values reported in this table are the mean and the standard deviation of each element. The overall classification accuracy values of these methods are shown in Table 8.

**Table 7:** The result of APM from the GLMs method with four different distributions

**GLM with**	**Actual class**	**Predicted class**			
		**STEMI**	**NSTEMI**	**UA**	**Others**
**Normal Dist.**	**STEMI**	86.82 ± 4.59	1.55 ± 2.11	11.64 ± 4.28	0.00 ± 0.00
	**NSTEMI**	60.52 ± 13.38	10.52 ±10.24	28.96 ± 8.36	0.00 ± 0.00
	**UA**	4.60 ± 2.17	12.16 ± 7.29	83.25 ± 7.23	0.00 ± 0.00
	**Others**	11.16 ± 11.28	7.76 ± 9.43	81.07 ±13.77	0.00 ± 0.00
**Inv. Gaussian Dist.**	**STEMI**	88.06 ± 4.47	0.5 ± 1.01	11.44 ± 4.44	0.00 ± 0.00
	**NSTEMI**	69.26 ± 8.25	2.06 ± 2.86	28.68 ± 8.19	0.00 ± 0.00
	**UA**	4.72 ± 2.03	17.42 ± 9.52	77.86 ± 9.44	0.00 ± 0.00
	**Others**	12.39 ±11.3	8.57 ± 11.27	79.04 ±15.53	0.00 ± 0.00
**Poisson Dist.**	**STEMI**	87.78 ± 4.35	0.69 ± 1.32	11.54 ± 4.29	0.00 ± 0.00
	**NSTEMI**	66.22 ±10.82	4.88 ± 6.70	28.90 ± 8.30	0.00 ± 0.00
	**UA**	4.82 ± 2.18	13.99 ± 7.73	81.19 ± 7.75	0.00 ± 0.00
	**Others**	12.23 ±11.21	7.83 ± 9.99	79.95 ±14.86	0.00 ± 0.00
**Gamma Dist.**	**STEMI**	87.78 ± 4.47	0.55 ± 1.1	11.67 ± 4.44	0.00 ± 0.00
	**NSTEMI**	68.62 ± 9.03	2.49 ± 3.79	28.89 ± 8.24	0.00 ± 0.00
	**UA**	4.77 ± 2.10	15.66 ± 0.66	79.57 ± 8.63	0.00 ± 0.00
	**Others**	12.24 ±11.22	7.80 ± 9.74	79.96 ±14.17	0.00 ± 0.00

**Table 8:** Overall classification accuracy values for GLMs with different distribution functions

**Distribution**	**Mean**	**Std.**
**Normal**	68.49	3.93
**Inverse Gaussian**	64.83	4.71
**Poisson**	66.73	3.99
**Gamma**	41.99	4.38

Table 8 shows that GLMs with normal distribution presented the best classification performance (i.e. 68.49± 3.93) among other distributions. However, Table 7 shows that most of the samples from NSTEMI class were misclassified into STEMI class. Furthermore, most of the samples from “others” class were misclassified into UA class. Only the samples of STEMI and UA classes were classified correctly with an acceptable probability rate. Table 9 presents the results of APMs obtained from the other aforementioned classification algorithms. To obtain these findings, the algorithms were repeated several times with different random selections of train-test or train-validation-test data based on the descriptions available in Table 3. For further clarification, for instance, the process of model-building for the MLP classifier was described in details.

**Table 9:** The APM for different classifier methods

**Classifier**	**Actual class**	**Predicted class**			
		**STEMI**	**NSTEMI**	**UA**	**Others**
**ANFIS**	**STEMI**	88.72 ± 4.82	8.14 ± 4.15	1.57 ± 1.80	1.57 ± 1.85
	**NSTEMI**	31.21 ± 9.05	40.04 ± 9.19	23.33 ± 7.33	5.42 ± 4.53
	**UA**	2.72 ± 1.91	17.43 ± 6.01	78.02 ± 5.93	1.84 ± 1.91
	**Others**	5.79 ± 9.30	22.69 ±16.86	67.22 ±17.83	4.30 ± 6.71
**7-nn**	**STEMI**	94.24 ± 3.22	4.20 ± 2.84	1.56 ± 1.52	0.00 ± 0.00
	**NSTEMI**	31.07 ± 7.64	47.14 ± 8.72	21.78 ± 7.41	0.02 ± 0.22
	**UA**	2.05 ± 1.32	3.64 ± 1.84	94.20 ± 2.23	0.11 ± 0.33
	**Others**	7.44 ± 7.79	7.28 ± 7.77	85.23 ± 10.31	0.05 ± 0.80
**Native Bayes**	**STEMI**	83.22 ± 4.78	2.59 ± 1.96	11.99 ± 3.94	2.19 ± 2.59
	**NSTEMI**	20.06 ± 6.32	47.59 ± 7.40	28.98 ± 7.35	3.36 ± 2.96
	**UA**	0.04 ± 0.20	7.16 ± 6.58	86.17 ± 7.23	6.64 ± 4.15
	**Others**	0.84 ± 2.88	7.85 ±10.09	80.99 ±12.58	10.32 ± 8.85
**ID3**	**STEMI**	84.55 ± 5.81	13.3 ± 5.61	1.92 ± 1.88	0.23 ± 0.73
	**NSTEMI**	26.59 ± 7.58	46.05 ± 9.00	25.09 ± 7.91	2.28 ± 2.99
	**UA**	0.94 ± 0.94	6.05 ± 2.84	88.08 ± 3.76	4.93 ± 2.64
	**Others**	3.09 ± 5.56	12.41 ± 10.70	78.63 ±13.60	5.87 ± 8.03
**Bagging-ID3**	**STEMI**	91.77 ± 3.94	6.54 ± 3.51	1.65 ± 1.73	0.03 ± 0.23
	**NSTEMI**	30.59 ± 7.41	46.93 ± 7.29	22.32 ± 6.85	0.16 ± 0.72
	**UA**	1.13 ± 0.85	3.70 ± 1.83	94.07 ± 2.36	1.09 ± 1.05
	**Others**	3.21 ± 4.87	9.84 ± 8.05	85.00 ± 9.50	1.95 ± 4.30
**RBF (7 neurons)**	**STEMI**	84.99± 4.98	1.76 ± 1.81	13.18 ± 4.74	0.07 ± 0.37
	**NSTEMI**	30.67±17.98	34.88 ±17.88	33.98 ±10.00	0.47 ± 1.31
	**UA**	2.34 ± 2.34	2.18 ± 1.73	95.41 ± 2.35	0.07 ± 0.27
	**Others**	5.40± 8.81	8.37 ± 9.10	84.88 ±12.10	1.34 ± 3.81
**MLP (9 neurons)**	**STEMI**	93.78 ± 5.05	2.67 ± 2.72	3.38 ± 4.07	0.17 ± 0.68
	**NSTEMI**	29.93 ±10.41	48.33 ±10.21	21.43 ± 9.20	0.31 ± 1.14
	**UA**	0.73 ± 1.26	2.98 ± 2.11	96.21 ± 2.55	0.08 ± 0.34
	**Others**	3.07 ± 6.56	9.76 ± 12.30	82.54 ±15.10	4.62 ± 9.64

In the MLP classifier, the seven selected features and the four class labels were considered as input and output nodes respectively. At first, an MPL with “N” hidden nodes was considered. In the next step, three-way data splits technique and also repeated random sub-sampling cross-validation method were used. This is, the data set was divided into training, validation, and test sets. The train set was used for determining optimal weights with back - propagation rules, while incorporating the validation set. Validation set was used to determine the optimal number of neurons in the hidden layer, as well as to avoid over-fitting (determine a stopping point for the back propagation algorithm). When the best “weights” were found, the performance of this network was calculated based on the classification error on the validation set. It should be mentioned that in order to make the final network unbiased, the train-validation-test process is repeated 100 times with different randomly selected starting values. Accordingly, the average of the 100 error values (based on the validation set) was considered as the final classification performance of the MLP with N hidden nodes. These steps were also done for different number of hidden nodes (from 2 to 13). At the end of this process, the best MLP having minimum average error value was determined as the final model.

As was mentioned earlier, the validation set was used to select the final model; consequently, in order to achieve unbiased error rate estimation of the final model the testing set was used. In fact, once final model was chosen, its real accuracy is assessed on the test set. The optimum number of hidden layer neurons was determined 9 for MLP.

The overall classification accuracy of all the methods is shown in Table 10. The MLP followed by the 7-NN method had the best classification performance with overall accuracies of 83.24 ± 3.17% and 82.92 ± 2.45%, respectively. It should be mentioned that the priority of the k - NN method over other classification methods (except MLP) may be due to this fact that, the k-NN classifier takes the advantage of the feature selection k-NN-based method.

**Table 10:** Overall classification accuracy of all the methods

**Classifiers**	**Accuracy (%)**	
	**Mean**	**Std.**
GLM with normal Dist.	68.49	3.93
ANFIS	71.31	3.73
7-NN	82.92	2.45
Naive Bayes	75.51	4.14
ID3	76.33	2.83
Bagging-ID3	81.12	2.43
RBF	78.42	3.59
MLP	83.24	3.17

As we expected, Bagging-ID3 generated better results than ID3 due to the fact that it is actually a modified version of ID3. The results of this classifier were close to MLP showing its capability in our data classification task. On the other hand, the performance of 7-NN classifiers was also very close to MLP. By looking at the best resulting APMs belonging to the MLP classifier, it can be concluded that, firstly, the samples of “others” class were very similar to the samples of UA class because most of these samples were misclassified as UA class. It should be noted that this problem was caused by the fact that the sample percentage of this class (or correspondingly its prior probability) was smaller than UA (see Table 5). However, this problem is not crucial because the risk of this misclassification is not harmful for the patient. Secondly, a large percentage of NSTEMI samples were misclassified as STEMI and UA classes. This problem may be caused by its low prior probability or its similarity to the classes, especially to the STEMI class. A misclassification of NSTEMI sample as STEMI class is not risky for the patients because the patients continue to remain under monitoring. However, being misclassified to the UA class could be harmful for the patient because the patient might be discharged. Nevertheless, this problem is not crucial in our MLP classifier.

Since the prior probability of classes we used are not the same, it is more appropriate to interpret the correctness of the classifier decision. This means that we should know the correct probability of a decision which assigns a sample to a class $c_{i}^{*}$. For this purpose, we can use CPM which is defined in this section. As mentioned earlier, each element (*i,j*)of CPM indicates the probability of a sample classified as $c_{i}^{*}$ , actually belonging to *c*_*i*_ or $\;p\;\left(c_{i}|c_{j}^{*}\right)$. The CPM of MLP classifier is presented in Table 11.

**Table 11:** The CPM of MLP method (with 9 neurons)

**Actual class**	**Predicted class**			
	**STEMI**	**NSTEMI**	**UA**	**Others**
**STEMI**	92.28	30.70	0.40	1.88
**NSTEMI**	1.50	28.32	0.94	3.42
**UA**	6.19	40.92	98.65	94.19
**Others**	0.03	0.06	0.01	0.51

It is observed that in UA class the 98.7% of the decisions were correctly made. Therefore, if the classifier assigns a patient to UA class, we should not worry about the risk that the patient has STEMI or NSTEMI. In other words, the risk of discharging an MI patient as a UA case was too low 1.35%.

**Figure 3:**
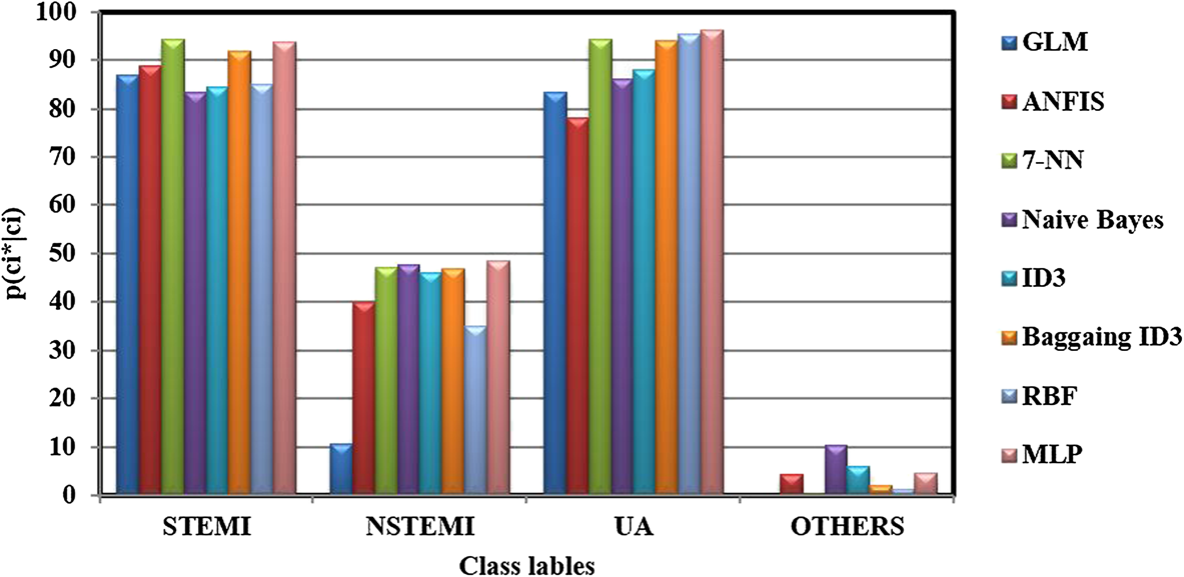
**Bar graph of diagonal elements of APM for all methods, each bar corresponds to the accuracy probability (i.e.**$p\left(c_{i}^{*}\left|c_{i}\right)\right)$**) of class *c***_***i***_**.**

**Figure 4:**
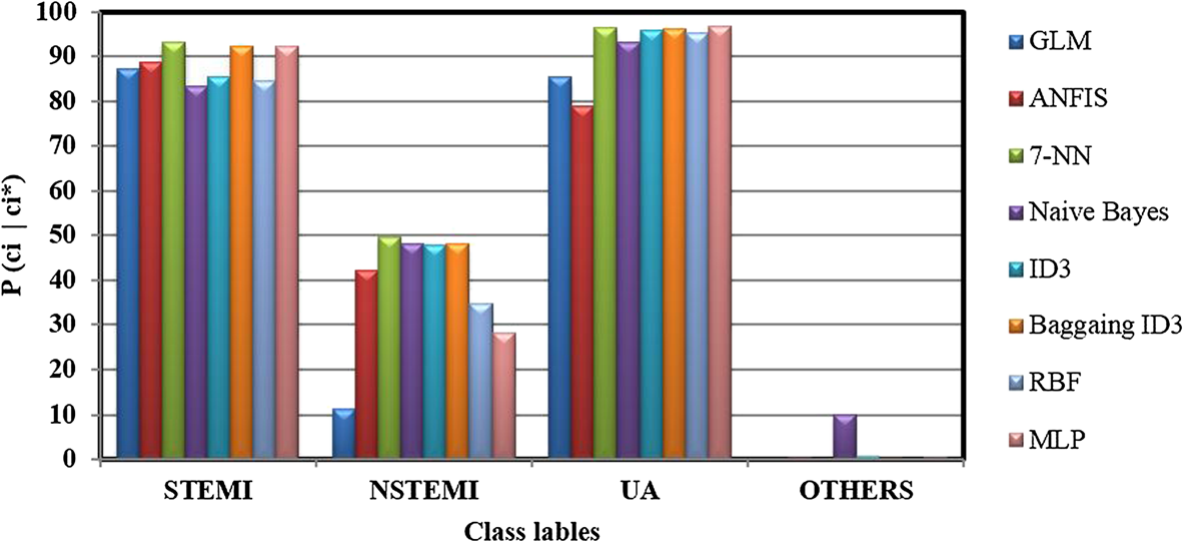
**Bar graph of diagonal elements of CPM for all methods, each bar corresponds to the correctness probability (i.e.**$p\left(c_{i}{\left|c\right)}_{i}^{*}\right)$) of class *c*_***i***_**.**

For a better comparison of accuracy and correctness between all methods studied in this research, the bar graphs of diagonal elements of all APMs and CPMs were shown in Figures 3 and 4, respectively. These Figures show the probabilities of $p\;\left(c_{i}^{*}|c_{i}\right)$ (See Figure 3) and $p\;\left(c_{i}|c_{i}^{*}\right)$ (See Figure 4). It can be seen that the performance of MLP classifier was significantly better than the rest. However, both accuracy and correctness measures for “others” and NSTEMI were not high enough which means that the classifier failed to model these regions of data. This problem can be solved by acquiring either more samples or new clinical features which can distinguish them more precisely.

## Conclusion

Accuracy improvement strategies play a key role in correctly classifying ACS patients, which ultimately saves valuable time and prevents potential misdiagnoses. Artificial intelligence-based approaches are powerful strategy, which can be used to this end. The current study proposed an integrated artificial intelligence-based method in order to discriminate among different types of ACS: UA, STEMI, and NSTEMI, with greater accuracy than current methods. A k-NN-based feature selection algorithm was used to find a subset of the features with the best classification accuracy. As a result, the feature numbers considerably reduced to only seven. Finally, eight different common pattern recognition methods were used to classify the subtypes of ACS based on the seven selected features. The performance of the classifiers was then compared based on their accuracy computed from their confusion matrices. The MLP and 7-NN methods showed the highest accuracy was 83.24% and 82.92%, respectively. The GLM and ANFIS methods, on the other hand, showed the lowest overall classification accuracy of 68.5% and 71.3%, respectively. Overall, MLP showed the best performance between these classifiers. Although MLP classifier is slightly more accurate than k-NN classifier, k-NN has some advantages such as simple implementability, understandability and interpretability; hence, future research is needed to further elucidate this model. In summary, early accurate classification of ACS by the incorporation of an AI-based feature selection with an AI-based classifier demonstrated promising results that can be used in the clinical field to timely diagnose and treat ACS patients.

## Competing interests

The authors declared that they have no competing interests.

## Authors’ contributions

In this study all authors have had key contribution and also, read and approved the final manuscript.
